# Editorial for a Special Issue of Selected Papers from the 23rd Annual Conference and 12th International Conference of the Chinese Society of Micro-NanoTechnology (CSMNT 2021)

**DOI:** 10.3390/mi14010020

**Published:** 2022-12-21

**Authors:** Qiyuan Feng, Shun Zhang, Qingyou Lu

**Affiliations:** 1 High Magnetic Field Laboratory, Hefei Institutes of Physical Science, Chinese Academy of Sciences, Hefei 230031, China; 2 Anhui Laboratory of Advanced Photon Science and Technology, University of Science and Technology of China, Hefei 230026, China; 3 Anhui Province Key Laboratory of Condensed Matter Physics at Extreme Conditions, High Magnetic Field Laboratory of Anhui, Hefei 230031, China; 4 Hefei Science Center, Chinese Academy of Sciences, Hefei 230031, China

The Annual Conference and International Conference of the Chinese Association of Micro-NanoTechnology is a comprehensive, cross-disciplinary, high-level academic conference that has been held annually since 1994 and has become an important academic event in the field of micro- and nanotechnology. The conference includes an opening ceremony, reports related to the main and sub-venues, education and training, a call for papers, and technical exhibits. Moreover, it provides a platform for domestic and foreign micro- and nanotechnology workers to exchange ideas, technology, and scientific research pertaining to micro- and nanotechnology and related fields.

This Special Issue contains sixteen papers and one review from the 23rd Annual Conference and 12th International Conference of the Chinese Society of Micro-Nano Technology (CSMNT 2021), which was held in Harbin, China (24–27 September 2021). The papers highlight new findings and technologies related to micro/nanoenergy, micro-electromechanical systems, nanosystems, and nanomaterials, as well as emerging related fields.

This issue includes a proposal for a novel spiral-wound optic-fibre sensor to monitor the corrosion of steel bars by some authors; the feasibility of the spiral distributed sensors was verified experimentally. This method can be used to evaluate the initial and final cracking behaviours of concrete structures, as well as steel bar corrosion [1]. Due to differences in fibre type and length, ambient temperature, and strain, fully distributed fibre optic sensors fail to locate damage accurately and cause greater error. The authors propose a new positioning method that combines fully distributed fibre optic sensors with fibre Bragg gratings, which enables the accurate localization of structural damage during long-term monitoring with fully distributed fibre optic sensors [2]. Using COMSOL Multiphysics software and a neural network/genetic algorithm approach, the authors optimized the process parameters of microscale meniscus-confined electrodeposition to achieve highly efficient, high-quality deposition. Simulations and experimental results showed that the maximum deposition rate error was only 2.0%. This will promote the application of microscale meniscus-confined electrodeposition for three-dimensional printing, picking up micro-components, and composite coating [3]. A more accurate rate-dependent piezoelectric actuator nonlinear model is needed for random signal dynamic tracking control systems, such as active vibration control. The authors propose a Hammerstein model based on a fractional-order rate correlation. By comparing a simulation and experimental data for multiple sets of sinusoidal excitations at different frequencies, the effectiveness of the proposed modelling method, and the accuracy and rapidity of the identification algorithm, were verified [4]. To solve installation errors occurring during the calibration of accelerometers, the authors propose a calibration method that uses a double turntable centrifuge. This method can eliminate the effect of installation errors in the angle and radius, and the static radius can be measured directly based on the model [5]. It can be used to calibrate accelerometers and has high potential for engineering applications.

Light-emitting diodes (LEDs) are widely used in medicine, navigation, and landscape lighting. The development of high-power LEDs requires the dissipation of LED heat. The authors reviewed the packaging technology and structure in terms of the thermal performance of LED packaging and introduced related technologies that promote heat dissipation in LED packaging [6]. Some authors created a finite element thermal model of an LED filament and used finite element steady-state analysis to compare several methods that affect heat dissipation of the LED filament package. They also calculated LED heat dissipation and lighting capacity through the application of LED-related theory [7]. Other authors deposited a layer of magnetic metal on silicon substrate by physical vapour deposition and then developed a 2-inch, 3,175-pixels-per-inch (PPI) magnetic metal hard mask on silicon substrate (MMS) using deep silicon etching and other micro/nanoprocessing for patterning organic light-emitting diode (OLED) displays, which can achieve smaller pixel sizes and more PPI [8].

Another group of authors used microelectromechanical systems (MEMS) combining electrical, optical, and mechanical components at the micro-scale for miniaturizing mechanical micro devices [9]. The authors propose a thermoelectric MEMS microwave power sensor with an inline self-detection function based on the principle of microwave power-heat-electricity; a voltage divider circuit was designed to realize a terminal load self-calibration function [10]. Oocyte penetration is an essential step for many biological technologies. Although the success rate of robotic cell penetration is very high, the development potential of oocytes after penetration was not improved significantly compared with manual operations. The authors optimized the oocyte penetration speed based on the intracellular strain [11]. The authors present a microfluidic system for high-resolution nanoparticle separation based on negative magnetophoresis; this may serve as a versatile tool for separating nanometric objects of environmental or biological importance, such as nanoparticles, viruses, and other biological agents [12]. The authors designed and fabricated a soft wireless passive pressure sensor, with a fully flexible Ecoflex substrate and multi-walled carbon nanotube/polydimethylsiloxane (MWCNT/PDMS) bilayer and pyramid dielectric structure. Based on the principle of a radio-frequency resonator, the device achieved pressure sensing with changeable capacitance [13]. To improve the cooling performance of the insulated gate bipolar transistor (IGBT) modules used in motor controllers, authors analysed the internal thermal resistance and external heat dissipation structure of the module and proposed three flow-channel structure models of water-cooled heatsinks [14].

Another group of authors designed and fabricated a laser-controlled intelligent initiation system with inherent safety and a laser-controlled explosion-initiating device (LCEID) using integrated safe-and-arm, electromagnetic pulse-resistant, and fast-acting technologies. A modular design and integrated circuit fabrication techniques were also used [15]. Some authors designed and fabricated an array piezoelectric–electromagnetic hybrid energy harvester and coupled the piezoelectric and electromagnetic energy by physically arranging permanent magnets on the cantilever beams, which gave the cantilever beam a customized resonant frequency and greater vibration amplitude under external excitation, thereby significantly improving the output power of the piezoelectric part [16]. Finally, some authors constructed a CdTe quantum dot-based fluorescent probe that can be used to determine felodipine content; there was no substantial difference between the results of the determination method and the labelled felodipine tablet contents. Therefore, this method can be used to determine the felodipine content of drugs and various substances accurately and reliably using simple reaction conditions that are easy to control [17].

We would like to thank all of the authors for contributing their original manuscripts to this Special Issue, and the reviewers for their participation in the peer-review process, which helped to improve the quality of the papers.
